# P-2145. Low Prevalence of Cytomegalovirus Infections Beyond Two Years after Solid Organ Transplant: An Opportunity for Diagnostic Stewardship

**DOI:** 10.1093/ofid/ofaf695.2308

**Published:** 2026-01-11

**Authors:** Madeleine R Heldman, Eileen K Maziarz, Jennifer Saullo, Patrick C Tam, Sonya Kothadia, Amanda Seidenfeld, Sana Arif, Julie M Steinbrink, Beatrice Sim, Jonathan Huggins, Matthew Ellis, Adam Devore, Joseph Lerman, M Christina Segovia, Lisa McElroy, Cameron R Wolfe, Barbara D Alexander, Arthur W Baker

**Affiliations:** Duke University, Durham, NC; Duke University Medical Center, Durham, NC; Duke University, Durham, NC; Duke University School of Medicine, Durham, NC; Duke University, Durham, NC; Duke Health, Durham, North Carolina; Duke University, Durham, NC; Duke University Medical Center, Durham, NC; Duke University Medical Center, Durham, NC; Duke University Hospital, Durham, NC; Duke University, Durham, NC; Duke University, Durham, NC; Duke University, Durham, NC; Duke University, Durham, NC; Duke University, Durham, NC; Duke University, Durham, NC; Duke University School of Medicine, Durham, NC; Duke University School of Medicine, Durham, NC

## Abstract

**Background:**

Solid organ transplant (SOT) recipients frequently undergo cytomegalovirus (CMV) PCR testing throughout their post-transplant course. While CMV infections are common early (< 2 years) after SOT, the prevalence of CMV infections beyond 2 years has not been reported.

**Figure 1:**
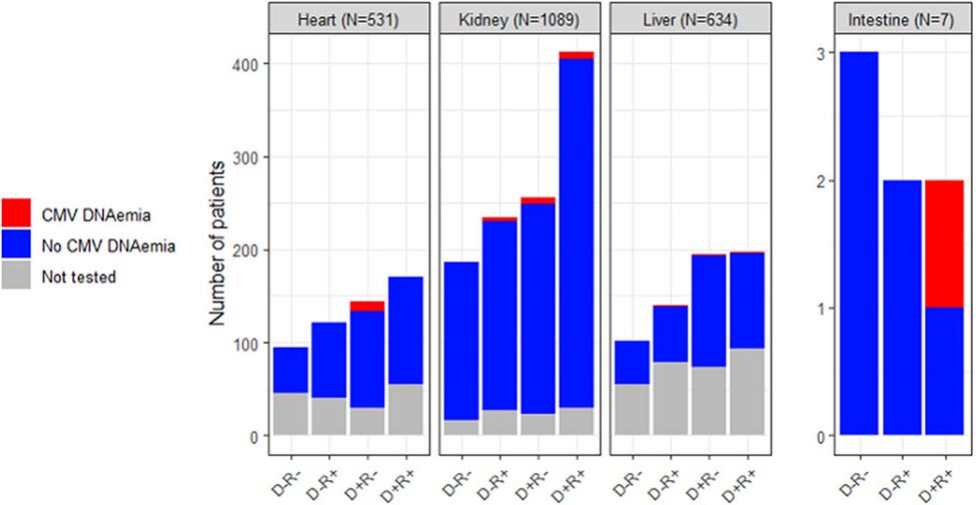
CMV PCR testing and CMV DNAemia beyond two years after transplant in solid organ transplant recipients who survived to two years

**Table 1:**
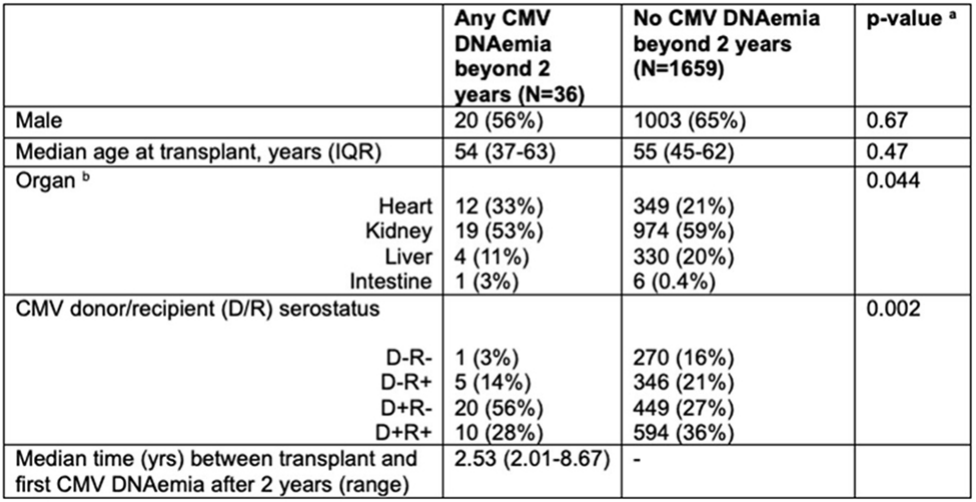
Solid organ transplant recipients who underwent CMV PCR testing at least once beyond two years after transplant a) Chi squared and Fisher exact tests were used for categorical variables and the Mann-Whitney U test was used for continuous variables. b) Heart includes 3 heart-liver recipients and 21 heart-kidney recipients. Kidney includes 61 kidney-pancreas recipients. Liver includes 54 liver-kidney recipients. Intestine includes 4 intestine-liver-pancreas recipients.

**Methods:**

We performed a single center retrospective study to determine the period prevalence of CMV PCR testing, CMV DNAemia, and CMV disease beyond 2 years after non-lung SOT. All non-lung, adult SOT recipients (SOTR) who underwent their first SOT between 2/24/14-8/1/22 and who survived through 2 years were included. Patients were followed until death, re-transplant, or 8/1/24 (whichever came first). CMV DNAemia was defined as any plasma CMV load ≥ 450 IU/mL, which was a common threshold to initiate preemptive CMV therapy at our institution. CMV PCRs were performed for evaluation of symptoms of CMV disease or as asymptomatic screening at clinician discretion. Patients did not receive CMV prophylaxis beyond 6 months, except for the 30-days following treatment with lymphodepleting antibodies for rejection.

**Figure 2:**
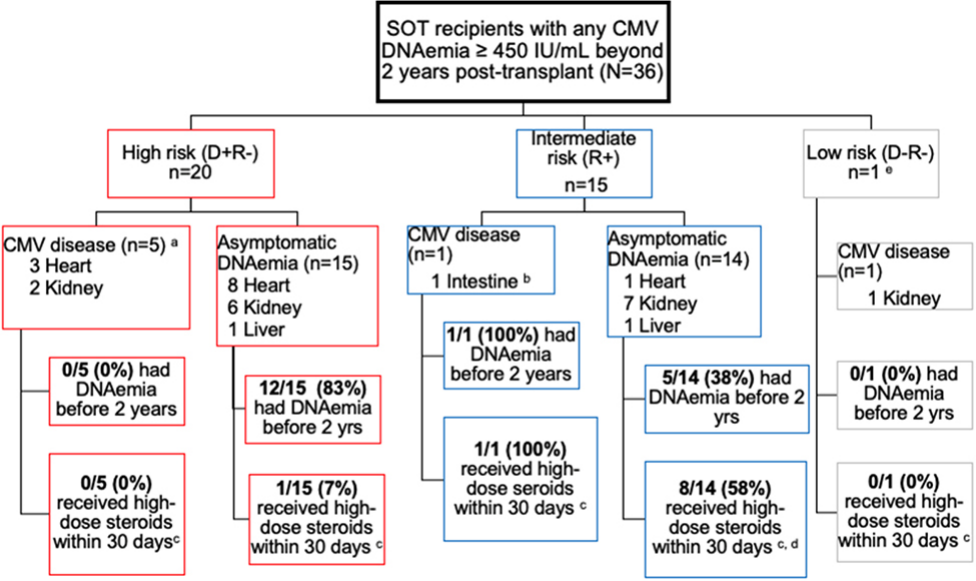
Solid organ transplant recipients with CMV infections beyond two years after transplant a) All cases of CMV disease in D+R- and D-R- consisted of CMV syndrome. b ) This seropositive recipient (R+) was an intestine-liver-pancreas recipient who developed proven CMV enteritis involving the small bowel allograft within two weeks after starting corticosteroids for treatment of rejection. c ) Patients were considered to have received high-dose steroids if they had received ≥ 40 mg/day of prednisone (or equivalent) for at least 7 days within the 30 days before the first occurrence of CMV ≥ 450 IU/mL beyond the 2-year post-transplant date. d) Two seropositive (R+) kidney recipients with asymptomatic CMV DNAemia who received high-dose steroids also received anti-thymocyte globulin within the previous 30 days. Both were taking valganciclovir prophylaxis that was underdosed for renal function at the time of initial CMV DNAemia. No other patients with CMV DNAemia received lymphodepleting antibodies within 30 days prior to their first CMV viral load ≥ 450 IU/mL. e) One case of CMV disease (CMV syndrome) in a D-R- kidney recipient was likely due to community acquired CMV infection

**Results:**

Of 2261 SOTR who survived to 2 years after their first SOT, 1695 (75%) underwent CMV PCR testing at least once in the period beyond 2 years, with a mean of 8 CMV PCRs per tested patient (range 1-85) during a mean follow-up time of 2.5 years (range 0.1-8.40) beyond the 2-year mark. CMV DNAemia occurred in 36/1695 tested patients (2.1%, Figure 1). Heart transplant and CMV donor positive/recipient negative (D+R-) status were associated with DNAemia (Table 1). Nine of 15 (60%) CMV seropositive (R+) SOTR with DNAemia received high-dose steroids within 30 days of DNAemia onset compared to 1/20 (5%) D+R- SOTR with DNAemia (Fisher exact p < 0.001). CMV disease occurred in 7 tested patients (0.4%), including five D+R- SOTR without prior CMV infection, one R+ patient who received recent high-dose steroids for rejection, and one CMV D-R- patient with primary, community-acquired infection (Figure 2).

**Conclusion:**

CMV infections were rare beyond 2 years after non-lung SOT. Late CMV disease occurred in a small number of CMV seronegative SOTR without prior CMV infection and one CMV seropositive SOTR recently treated with high-dose steroids. However, CMV PCR testing beyond the 2-year mark likely has low diagnostic yield for most non-lung SOTR.

**Disclosures:**

Madeleine R. Heldman, MD, MS, Karius, Inc: Advisor/Consultant Jennifer Saullo, MD, Pharm D, RMEI Medical Education: Honoraria|UpToDate: Royalties Julie M. Steinbrink, MD, MHS, Biomeme: patents for gene expression classifiers of fungal infection|McGraw Hill Publishing: royalties

